# Transcriptomic analysis of pea plant responses to chitooligosaccharides’ treatment revealed stimulation of mitogen-activated protein kinase cascade

**DOI:** 10.3389/fpls.2023.1092013

**Published:** 2023-03-08

**Authors:** Polina Yu. Kozyulina, Olga A. Pavlova, Elizaveta S. Kantsurova (Rudaya), Andrey D. Bovin, Svetlana A. Shirobokova, Aleksandra V. Dolgikh, Alina M. Dymo, Elena A. Dolgikh

**Affiliations:** Laboratory of Signal Regulation, All-Russia Research Institute for Agricultural Microbiology, St.-Petersburg, Russia

**Keywords:** RNAseq, chitooligosaccharides, signal transduction, MAP kinase signaling cascade, gene expression regulation, pea *Pisum sativum* L.

## Abstract

Since chitooligosaccharides (COs) are water-soluble, biodegradable and nontoxic compounds, their application may be considered as a promising plant-protecting agent. However, the molecular and cellular modes of action of COs are not yet understood. In this study, transcriptional changes in pea roots treated with COs were investigated *via* RNA sequencing. Pea roots treated with the deacetylated CO8-DA at low concentration (10^-5^ М) were harvested 24 h after treatment and their expression profiles were compared against medium-treated control plants. We observed 886 differentially expressed genes (fold change ≥ 1; p-value < 0.05) 24 h after treatment with CO8-DA. Gene Ontology term over-representation analysis allowed us to identify the molecular functions of the genes activated in response to CO8-DA treatment and their relation to biological processes. Our findings suggest that calcium signaling regulators and MAPK cascade play a key role in pea plant responses to treatment. Here we found two MAPKKKs, the PsMAPKKK5 and PsMAPKKK20, which might function redundantly in the CO8-DA-activated signaling pathway. In accordance with this suggestion, we showed that *PsMAPKKK* knockdown decreases resistance to pathogenic *Fusarium culmorum* fungi. Therefore, analysis showed that typical regulators of intracellular signal transduction pathways involved in triggering of plant responses *via* CERK1 receptors to chitin/COs in *Arabidopsis* and rice may also be recruited in legume pea plants.

## Introduction

The capacity of a plant to prevent pathogen attack can be defined by regulation of plant immunity. Plants have well-developed mechanisms regulating immunity and leading to activation of resistance to pathogens. These mechanisms are defined according to the pathogen molecules recognized by the plant. One of mechanism, known as microbe-associated molecular patterns (MAMPs)-triggered immunity (MTI), is based on the ability of plants to recognize MAMPs by cell surface transmembrane pattern recognition receptors (PRRs) (Zipfel, 2008; Dodds and Rathjen, 2010). Ligand perception by PRRs triggers intracellular signal transduction followed by plant defense responses activation and restriction of pathogen distribution. Despite the wide variety of MAMPs, plants demonstrate pretty similar responses to recognition of these diverse structures, based on activation of intracellular receptor-like cytoplasmic kinases and mitogen-activated protein kinases (MAPKs) triggering defense responses in the host. Among them, the MAPK cascade is highly conserved and involved in phosphorylation of various downstream protein targets, which leads to the activation of defense responses. In general, MAPK cascade consists of three protein kinases: a MAP kinase kinase kinase (MAPKKK), a MAP kinase kinase (MAPKK), and a MAP kinase (MAPK), which are involved in sequential activation by phosphorylation (Chen et al., 2021). In the model plant *Arabidopsis*, 21 MEKK1-type MAPKKKs, 10 MAPKKs and 20 MAPKs were identified (Ichimura et al., 2002).

To overcome the plant immune responses, pathogens produce a variety of effectors entering the host cells. The effectors are able to suppress plant immune response, targeting PRRs and downstream signaling regulators, allowing the pathogens to infect the plant (Ichimaru et al., 2022). In response plants have developed an effector-induced immune system referred to as effector-triggered immunity (ETI) (Dodds and Rathjen, 2010). The intracellular receptor proteins with nucleotide-binding and leucine rich domains (NBS-LRR or NLRs) were shown to be involved in the recognition of effectors and triggering plant resistance. In the absence of a specific NBS LRR to the effector, the plant became susceptible to microbial infection.

Derivatives of chitin and chitosan known as chitooligosaccharides (COs) are well-known MAMPs showing the ability to trigger resistance in plants by elicitation of defense responses. COs are defined as chitin or chitosan oligomers consisting of N-acetyl-D-glucosamine or D-glucosamine residues with a degree of polymerization less than 20 and molecular weight of up to 3900 Da (Jeon, 2001). Their application results in MTI-triggering resistance to a broad range of plant pathogens. These compounds act as plant-protecting agents and may be considered as safe and environmentally friendly agrochemicals. However, the influence of these compounds has been investigated mainly in model plants such as *Arabidopsis* and rice, while the mechanisms triggered by COs and operating in crop plants remain little studied.

Plants have a highly sensitive perception system for chitin, chitosan and COs as recent studies have shown (Kaku et al., 2006; Miya et al., 2007; Shimizu et al., 2010; Willmann et al., 2011; Liu et al., 2012; Cao et al., 2014; Shinya et al., 2014). These proteins belong to a specific class of PRRs receptors having Lysin motif (LysM) in extracellular domains. In *Arabidopsis* and rice, the Chitin Elicitor Receptor Kinase 1 (CERK1) receptors can recognize more than one elicitor molecules with the common elicitor motifs such as chitin/COs as well as peptidoglycan and its derivatives. These multifunctional receptors play a crucial role in plant resistance to pathogen attack (Miya et al., 2007; Liu et al., 2012; Wan et al., 2012). However, the precise mechanisms regulating signal transduction in CERK1-triggered immunity are still far from understanding. The pea (*Pisum sativum* L.) is one of the most important crops worldwide, however, the molecular and cellular modes of action of COs in this plant remain poorly characterized and require further study. Deacetylated oligomers such as heptamers and octamers (CO7-DA, CO8-DA) are the most effective elicitors inducing the accumulation of phytoalexins and synthesis of pathogenesis-related proteins in pea (Kendra and Hadwiger, 1984; Kendra et al., 1989; Hadwiger, 1994; Hadwiger, 2015). In accordance with this, we have shown recently a high affinity of CERK1-like receptor in legume plant pea *Pisum sativum* L., the PsLYK9, to CO7-DA and CO8-DA molecules (Leppyanen et al., 2021). Here we focused on transcriptomic analysis of CO8-DA-triggered immunity through PsLYK9 receptor in pea *Pisum sativum* L. and found a set of putative intracellular regulators, which may be involved in signal transduction and activation of plant responses to this elicitor.

## Materials and methods

### Plant material

Pea *Pisum sativum* L. cultivars Frisson and Finale were used for experiments. Pea seeds were sterilized with sulphuric acid for 5 min, washed 5 times with water, transferred on 1% water agar plates and germinated at room temperature in the dark. Four to five-day-old seedlings were used for treatment with elicitors or *Agrobacterium*-mediated transformation.

### Plant treatment with chitooligosaccharides

Deacetylated COs with main degree of polymerization around eight (CO8-DA): Mn = 1089, Mw = 1514, Ip = 1.39, CDS = 93% in Cl-form (The Center of Bioengineering Russian Academy of Science, Moscow, Russia), were used for treatment. Four- to five-day-old seedlings of cv. Frisson were placed on the supporting foil surface in glass jars with 0.5x Jensen’s medium (control) (van Brussel et al., 1982) or 0.5x Jensen’s medium with CO8-DA (10^-5^ M) and incubated for 24 h. After treatment, the roots of pea seedlings were harvested for RNA extraction and gene expression analysis and immediately frozen at −80°C.

The substrate used for experiments with composite pea plants infected with pathogenic fungus *Fusarium culmorum* was 8–10 mm sterilized vermiculite. Two strains of phytopathogenic fungi *F. culmorum* (Wm. G. Sm.) Sacc. (weakly aggressive 891 and highly aggressive 334 strains) were used to infect pea plants. To obtain inoculum, *F. culmorum* 891 or 334 strains were grown on Chapek’s agar for 10–14 days and then washed from the plates with sterile water. Composite plants cv. Finale were placed into 250 ml plastic vessels with vermiculite saturated with Jensen’s medium and containing 3 x 10^5^ conidia of the pathogenic fungus *F. culmorum* 891 or 334 strains. Plants were grown in a growth chamber at 21°C in 16 h light/8 h dark cycles, at 60% humidity. The intensity of plant infection with phytopathogenic fungi was carried out according to the method proposed earlier (Leppyanen et al., 2017).

### Plasmid constructs

Full-length encoding sequences for *PsMAPKKK5* (Psat5g044960), *PsMAPKKK20* (Psat4g086600) and *PsMAPKK1* (Psat1g131480), *PsMAPKK2* (Psat4g016480), *PsMAPKK3* (Psat1g054880), *PsMAPKK4* (Psat7g263320), *PsMAPKK5* (Psat1g050720), *PsMAPKK6* (Psat1g029480) were amplified by PCR from cDNAs prepared from roots of wild type pea plants cv. Finale using Phusion Flash High-Fidelity PCR Master Mix (Thermo Fisher Scientific, Waltham, MA, USA) and specific primers (**Supplementary Table 1**) and ligated into pENTRY/D-TOPO or pDONR221 vectors (Thermo Fisher Scientific, Waltham, MA, USA). For the *PsMAPKKK20* RNAi construct, the 328 bp DNA fragment corresponding to +162 to +489 region of *PsMAPKKK20* cDNA was amplified by PCR using specific primers (**Supplementary Table 1**), and transferred using the Gateway system with BP clonase to pDONR221 vector. At the next stage LR clonase reaction from pDONR221 into pK7GWIWG2 II Red Root vector was performed. The verified construct was transferred into the *Agrobacterium rhizogenes* ARqua 1.

For co-immunoprecipitation the full-length encoding sequences of *PsMAPKKK5, PsMAPKKK20* and *PsMAPKK1, PsMAPKK2, PsMAPKK3, PsMAPKK4, PsMAPKK5, PsMAPKK6* were cloned into pRSETa vectors with the sequences encoding 6xHIS or 3xFLAG using primers containing restriction sites. Electrocompetent *E. coli* C41 were transformed with constructs in pRSETa-6xHIS or pRSETa-3xFLAG vectors using Gene Pulser XCell electroporation system (Bio-Rad Laboratories, CA, USA) and grown on selective LB medium. A few fresh colonies were transferred into a big flask containing 200 ml LB with ampicillin 100 µg/ml and culture was grown up to OD600 = 0.6 at 37°C and then for 2 h at 28°C in presence of 0.5 mM IPTG with intensive shaking. The cell’s pellets were used for protein extraction or stored at -80°C.

### Сo-immunoprecipitation assay

Co-immunoprecipitation was carried out using a µMACS kit (Miltenyi Biotec, Bergisch Gladbach, Germany) containing the MicroBeads with immobilized anti-HIS and anti-DYKDDDDK (also known as FLAG-tagged) antibodies. The pellet of *E. coli* cells was resuspended in lysis buffer (50 mM tris-HCl pH 8.0, 1% Triton X-100, 150 mM NaCl) containing 1× complete protease inhibitor cocktail (INHIB1, Merck, Darmstadt, Germany) and cell lysate was obtained *via* the sonication (3 × 20 s). These lysates containing HIS-tagged (PsMAPKKK5, PsMAPKKK20) or FLAG-tagged (PsMAPKK1, PsMAPKK2, PsMAPKK3, PsMAPKK4, PsMAPKK5, PsMAPKK6) proteins were co-incubated with MicroBeads for 1 – 1.5 h on ice and then were loaded onto a µMACS column placed in the magnetic stand of a µMACS separator. Then, the column with associated proteins was washed with a lysis buffer 3 - 4 times. After elution with a denaturing elution buffer (50 mM tris-HCl (pH 6.8), 50 mM DTT, 1% SDS, 1 mM EDTA, 0.005% bromophenol blue, 10% glycerol), the precipitated proteins were analyzed by sodium dodecyl sulfate polyacrylamide gel (SDS-PAGE). In our investigation, we used two approaches to study co-immunoprecipitation using a µMACS kit. In the first variant, we passed FLAG-tagged proteins and HIS-tagged proteins after co-incubation through magnetic MicroBeads with anti-HIS antibodies. In another variant, proteins were passed through the column with MicroBeads with immobilized anti-FLAG antibodies.

### *Agrobacterium*-based plant transformation

The transformation of pea seedlings cv. Finale was performed as previously described (Leppyanen et al., 2019) with minor modifications. Seedlings at the stage of one internode were transformed with freshly grown *A. rhizogenes* strain ARqua 1 and placed on Jensen’s medium (3 – 4 plants per box) in round plastic boxes with green filter (E1674, Duchefa, The Netherlands). When callus had been formed in 10 – 12 days, the plants were transferred to Emergence medium with 150 µg/ml and incubated for 3 – 4 days. Composite pea plants were transferred into pots with vermiculite saturated with Jensen’s medium. Inoculation with rhizobial strain *Rhizobium leguminosarum* bv. *viciae* RIAM1026 was performed in 3 days after planting.

### RNA isolation

To isolate the RNA from the frozen roots, the tissues were ground with mortar and pestle using liquid nitrogen. Extraction of RNA from the roots was done using the PuroZOL RNA isolation reagent (BioRad Laboratories, California, USA). DNA was eliminated using DNAse digestion (Thermo Fisher Scientific, USA). RNA quality and quantity were determined by Nanodrop (Implen GmbH, Munich, Germany).

### RNA sequencing

The sequencing library was prepared following the guidelines for NEBNext mRNA magnetic isolation module purification kit (E7490) (https://www.neb-online.de/en/art/E7490) and NEBNext Ultra II directional RNA library prep kit for Illumina (E7760) (https://www.neb-online.de/en/art/E7770S) (New England Biolabs, USA). The quality of RNA was estimated using Bioanalyzer 2100 (Agilent Technologies, California, USA) prior to sequencing. The NEBNext Multiplex Oligos primer set for Illumina (Index Primers Set 2) (E7500S) (New England Biolabs, USA) was used for preparation. At all stages, the material was cleaned using AMPure XP beads (Beckman Coulter, USA). The sequencing was conducted with an Illumina NovaSeq 6000 (Illumina, San Diego, CA, USA) with all 6 samples on one NovaSeq lane, resulting in 25-30 million 150-bp-reads per sample.

### Data analysis

The analysis was started from raw sequencing data in fastq format. Two groups of libraries representing at least three independent biological repeats in both control and treated variants (3 - 4 plants for one replication), were sequenced on a NovaSeq 6000 (Illumina, San Diego, CA, USA) in paired-end 2 x 150 bp reads mode according to the manufacturer’s protocol. The resulting reads were trimmed off Illumina adapters, additionally, read ends were trimmed by sequencing quality (phred33 score <20) using Trimmomatic (Bolger et al., 2014). To obtain read counts per gene fastq files were further processed with RSEM package (Li and Dewey, 2011) using *Pisum sativum* genome v1a (Kreplak et al., 2019) as a reference and bowtie2 aligner (Langmead and Salzberg, 2012). For visual exploration of the data and heatmap representation, the obtained read counts were normalized using variance stabilizing transformation function of DESeq2 R package (Love et al., 2014), which normalizes raw count data with respect to library size and transforms it into a matrix of counts with constant variance along the range of mean values. A visual inspection of the samples using principal component analysis (PCA) was followed by differential expression analysis on a raw count matrix of 6 individual biological replicas.

### Differentially expressed genes

Data normalization and differential expression analysis were performed using R package “DESeq2” (Love et al., 2014). The data was divided per comparison into two groups: COs *vs.* Jensen’s medium (control). Genes having adjusted p-value < 0.05 and absolute log2 fold change ≥ 1 were considered significantly differentially expressed.

Heatmaps of the top differentially expressed genes were created using “gplots” R package (Warnes et al., 2022). Gene ontology (Ashburner et al., 2000) gene set enrichment analysis was executed using the GSEABase R package (Morgan et al., 2022) with cutoff values: odds ratio > 2, and p-value < 0.05.

Heatmaps of the top differentially expressed genes were created using “gplots” R package (Warnes et al., 2022) with cutoff values: log2 fold change > 2, and p-value < 0.05, genes for heatmaps representing individual pathways for better readability were selected based on cutoff p-value < 0.1 from the results of differential expression analysis. Gene ontology (Ashburner et al., 2000) gene set enrichment analysis was executed using the GSEABase R package (Morgan et al., 2022) and GOstats R package (Falcon and Gentleman, 2007) with cutoff values: odds ratio > 2, and p-value < 0.05.

### Real-time qPCR

After extracting the RNA as described above, first strand cDNAs were synthesized from 1 µg of total RNA using M-MLV Reverse Transcriptase (200 U/µL) (Thermo Fisher Scientific, USA) following the manufacturer’s instructions. Highly specific primers were designed using Vector NTI with melting temperatures (Tm) between 54 and 58°C and amplicon lengths of 100-300 bp. As references, the housekeeping gene of the Ubiquitin (Ubiq) was used.

The qPCR cycler was a CFX96 Touch Real-Time PCR Detection System (Bio-Rad Laboratories, Hercules, CA, USA), with initial denaturation at 95°C for 3 min followed by 45 cycles of 95°C for 30 s, 54 - 58°C for 40 s and 72°C for 30 s. Melting curve analysis was performed from 54 to 95°C, where the temperature increased by 0.5°C every 5 s. The total volume was 10 μl per sample, containing 1 μl of cDNA and 5 μl of 2xiQ SYBR Green qPCR Supermix (Bio-Rad Laboratories, Hercules, CA, USA).

### MAPK3/6 phosphorylation assay

Plant treatment was done with 10^-5^ M CO8-DA as elicitor for 15 min and 0.5x Jensen’s medium as a control in glass jars. Total root protein was isolated in a buffer containing 100 mM MES-KON (pH 6.8), 10 mM MgCl_2_, 1% sucrose, 0.1% Triton X-100, 1 mM DTT, 0.5 mM PMSF, 1× complete protease inhibitor cocktail (INHIB1, Merck, Darmstadt, Germany). Western-blots were done to visualize the phospho-MAPK3/6 and tubulin bands using anti–phospho-p44/42 MAPK and anti–α/β-tubulin antibodies (Cell Signaling, Danvers, MA, USA).

### A phylogenetic tree construction

A phylogenetic tree was constructed using the Maximum Likelihood method with help of the IQ-TREE tool v.1.6.1 (Nguyen et al., 2015) and with automatically determined substitution model using ModelFinder (Kalyaanamoorthy et al., 2017), Tree was based on amino acid sequences that were aligned using the MAFFT tool v7.453 (Katoh and Standley, 2013) with default settings. The bootstrap values were obtained from 1000 bootstrap replicates using Ultrafast Bootstrap Approximation (Minh et al., 2013).

## Results

### Transcriptomic analysis of pea roots treated with deacetylated chitooligosaccharides (CO8-DA)

**Figure 1:**
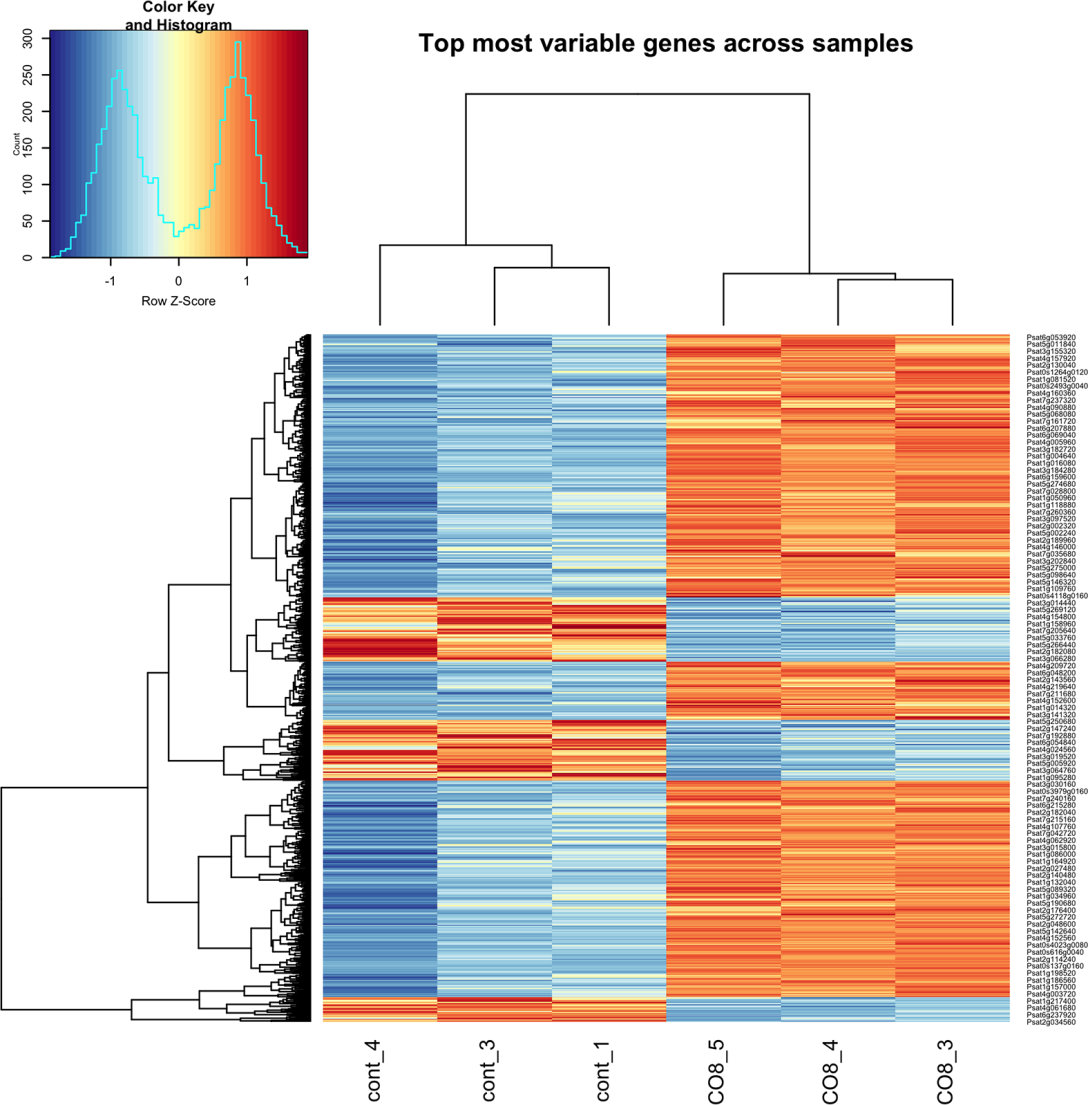
Differentially expressed genes (DEGs) in a cluster-wise heatmap of 6 biological replicas 24 h after CO8-DA treatment (Log2 fold change ≥ 1, p-value < 0.05).

In the current work, we compared the transcriptomes of CO8-DA-treated pea *P. sativum* L. plants with plants incubated in medium without elicitors. In our experiments around 886 genes were found to be differentially expressed (Log2 fold change ≥ 1; p-value < 0.05) 24 h after treatment of pea plants with deacetylated chitooligosaccharides CO8-DA (see Material and methods). Among these genes, 693 genes were up-regulated in CO8-DA-treated samples, while 193 genes were detected as down-regulated (**Figure 1** and **Supplementary Figure 1**). **Figure 1** shows the clustering of the differentially expressed genes based on their similar expression patterns. In all sample groups (CO8-DA versus control without treatment) the larger cluster represents the upregulated differentially expressed genes (**Figure 1**).

Gene Ontology term over-representation analysis was performed in order to check which molecular functions (**Supplementary Figure 2**) and biological processes (**Supplementary Figure 3**) are significantly activated in response to CO8-DA treatment. The highest rate of change was detected in chitinase and peroxidase activity, polysaccharide and phospholipid binding. We could also see the activation of protein kinases and calcium-dependent signaling and subsequent transcription factor activation (**Supplementary Figure 2**). Changes in biological processes were represented by over-representation of genes involved in cell death regulation and response to stress, such as wounding and external biotic stimulus, immune response and metabolic processes involved in defense mechanisms, such as and phenylpropanoid and flavonoid biosynthesis, chitin and cell wall polysaccharide metabolic processes, and hormone regulation mechanisms, such as response to salicylic acid (**Supplementary Figure 3**).

**Figure 2:**
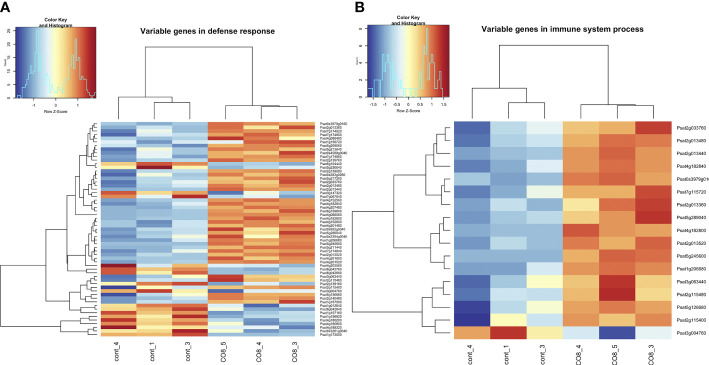
Heatmap of gene expression levels after CO8-DA treatment (3 biological replicas) in comparison to control (Jensen’s medium) (3 biological replicas). The list of genes that changed expression levels and were annotated in Gene Ontology terms as a part of “defense response” **(A)** and “immune system process” **(B)** biological processes in pea roots 24 h after treatment.

In accordance with the suggestion that in pea through CERK1-like receptor to COs, the LYK9, a complex of plant defense reactions was activated as one of the main reactions to treatment, we observed the up-regulation of genes encoding WRKY (Psat7g050880, Psat2g054280, Psat5g068680, Psat5g079680, Psat5g032760, Psat1g161840, Psat3g118040, Psat3g160600, Psat3g019520, Psat3g123560) and MYB (Psat1g209120, Psat7g159800) transcription factor family proteins, as well as C2H2 type zinc finger (Psat6g068960, Psat6g069040, Psat6g069000, Psat3g049400) transcription factor family protein (**Figure 2** and **Supplementary Table 2**). In addition, the increased level of transcripts encoding LysM-receptor-like protein LYR4 (Psat2g024920) was revealed. This co-receptor was previously shown to be involved in the regulation of defense responses to CO8-DA through PsLYK9/PsLYR4 complex formation in pea plants (Leppyanen et al., 2021). Moreover, differentially expressed genes encoding phenylalanine ammonia-lyase 1 and 2 (PAL1 (Psat1g046920) and PAL2 (Psat3g023040)), isoflavone synthase (Psat7g117880), peroxidases (Psat6g159360, Psat2g068760, Psat6g099960) and chitinases (Psat5g291720, Psat5g020240, Psat1g084840, Psat6g194680) were also found in pea root transcriptome. They belong to typical pathogenesis related marker genes, which are activated during plant resistance stimulation. Up-regulation of genes encoding PAL1 and PAL2 and isoflavone synthase (IFS) is consistent with the suggestion that these enzymes are involved in the biosynthesis of phytoalexins and other secondary metabolites. To verify the results of RNA-seq analysis, the expression of several genes was also estimated by real-time qPCR (**Supplementary Figure 4**). The significant stimulation of genes encoding PAL1 (Psat1g046920), IFS (Psat7g117880), LysM-receptor-like protein LYR4 (Psat2g024920), WRKY (Psat7g050880, Psat2g054280, Psat3g019520) was confirmed by real-time qPCR (**Supplementary Figure 4**). High number of upregulated defense-related genes may indicate that CO8-DA induces a strong plant disease resistance in a short period after treatment.

Plant treatment with CO8-DA resulted in stimulation of processes related to a cell death response, as it is evidenced by the activation of genes encoding cysteine/histidine-rich C1 domain proteins (Psat3g183160, Psat3g182680) and a whole complex of NBS-LRR proteins or NLRs (Psat4g182840, Psat2g140480, Psat7g174920, Psat7g174880, Psat7g174840, Psat5g289040, Psat2g139760, Psat0s3979g0160) (**Supplementary Table 2**) (Dodds and Rathjen, 2010; Hwang et al., 2014). These findings suggest that stimulation of expression of these genes may be sufficient to trigger early defense responses, such as the hypersensitive response.

One of the most important aims during screening of differentially expressed genes may be searching for the genetic markers responsible for plant resistance to pathogens, since these genes can be the subject of direct genetic approaches to increase plant crop stability. Here we have found several genes encoding disease resistance proteins (**Figure 2** and **Supplementary Table 2**). The gene Psat7g105600 encoding a probable homologue of LRR receptor-like serine/threonine-protein kinase At5g15730 is most likely a key candidate gene in triggering pea plant resistance. In *Arabidopsis* this gene is up-regulated and may be used as a marker of plant resistance to pathogen treatment (Kemmerling, 2011). The other gene Psat4g088560 encodes probable homologue of disease resistance protein At5g66900, which is important for regulation of *Arabidopsis* plant resistance to fungi (Saile et al., 2020).

Among the differentially expressed genes, a few genes (Psat1g029320, Psat2g044920) encoding Avr9/Cf-9 rapidly elicited proteins were also found (**Figure 2** and **Supplementary Table 2**). These genes confer resistance to the fungal pathogen *Cladosporium fulvum* (Cf) through recognition of secreted avirulence (Avr) peptides. The gene Psat3g098040 encodes probable receptor-like serine/threonine-protein kinase At1g18390 involved in a positive regulation of abiotic stress response *via* abscisic acid signaling (Shin et al., 2015). The information of their homologous genes in *Arabidopsis* might provide deeper understanding of functions of these genes during resistance development.

Enhanced level of the Psat7g177240 and Psat7g177200 transcripts encoding lignin-forming anionic peroxidases was identified in treated pea roots (**Supplementary Table 2**). In plants Casparian strip membrane domain proteins were suggested to be crucial for the assembly and activity of lignin biosynthetic enzymes. The lignin polymer deposition determines tolerance to abiotic stresses *via* modulating nutrient uptake and pathogen distribution prevention (Yang et al., 2015). Therefore, it suggests that plant treatment with CO8-DA elicitor may stimulate a complex of genes involved in lignin deposition as response to biotic and abiotic stresses.

### Hormonal regulation in plants as a response to CO8-DA treatment

**Figure 3:**
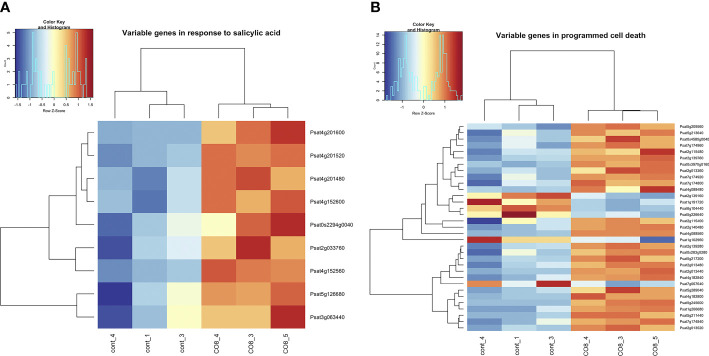
Heatmap of gene expression levels after CO8-DA treatment (3 biological replicas) in comparison to control (Jensen’s medium) (3 biological replicas). The list of genes that changed expression levels and were annotated in Gene Ontology terms as a part of “response to salicylic acid” **(A)** and “programmed cell death” **(B)** biological processes in pea roots 24 h after treatment.

Here, among differentially expressed genes we have identified the enrichment of genes belonging to the “response to salicylic acid” and “programmed cell death” pathways (**Figure 3** and **Supplementary Table 2**). Our transcriptomic analysis revealed that a number of genes related to jasmonic acid (JA) and salicylic acid (SA) signaling events and required for plant defense response development showed different expression levels between control and CO8-DA- treated pea roots. Among them, DCD (development and cell death) domain protein B2 (Psat7g024720), nematode resistance HSPRO2-like protein (Psat2g033760), patatin-like phospholipase (Psat5g043960), calmodulin-binding-like protein (Psat1g036680), heavy metal-induced protein 6B (Psat4g016000), probable serine/threonine-protein kinase Cx32 (Psat2g149240), serine/threonine-protein kinase RIPK-like (Psat1g133080), tubby-like C 2 protein (Psat7g228880) were observed. The Psat0s802g0040 encodes NIM1-interacting 1-like (NIMIN1) protein, which *via* physical interaction with Nonexpresser of PR genes (NPR1) regulates the signal transduction pathway leading to systemic acquired resistance (Weigel et al., 2005). Homologous proteins may function as positive regulators of plant cell death and SA-dependent defense responses in plants (Hwang et al., 2014).

We also identified the enhanced expression of genes encoding several transcription factors which had been reported to SA and JA mediated signaling pathways such as NAC-like transcription factor (Psat5g058640), MYB transcription factor (Psat4g080720), AP2/ERF domain transcription factor (Psat7g124720), AP2 domain class transcription factor (Psat5g063960) and others.

Ethylene-mediated signaling pathway is activated by CO8-DA. Among of ethylene-induced genes, the Psat3g176000 and Psat5g140800 encoding putative calcium-binding protein CML19 and calcium-binding protein CML38-like as well as Psat3g176000 and Psat2g042000 encoding putative calcium-binding protein CML19 and calcium-binding protein CML24 were found.

Furthermore, the expression of genes presumed to be involved in the auxin transport and response was also changed in roots treated with CO8-DA. The analysis showed enhanced level of Psat5g267280 and Psat0s1162g0080 transcripts encoding calcium-binding PINOID-binding protein 1 (PBP1-like) involved in auxin transport and SAUR-like auxin-responsive family proteins (Psat0s2933g0120) involved in response to auxin stimulus.

These results demonstrate that CO8-DA may essentially affect the hormonal status of pea plants.

### Intracellular regulators of signal transduction pathways

**Figure 4:**
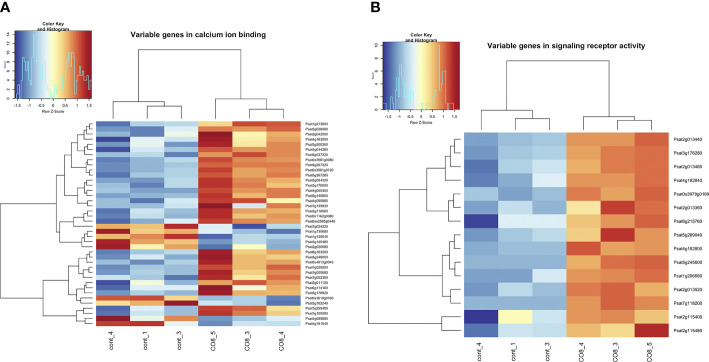
Heatmap of gene expression levels after CO8-DA treatment (3 biological replicas) in comparison to control (Jensen’s medium) (3 biological replicas). The list of genes that changed expression levels and were annotated in Gene Ontology terms as a part of “calcium ion binding” **(A)** and “signaling receptor activity” **(B)** biological processes in pea roots 24 h after treatment.

Among differentially expressed genes we have identified the enrichment of genes belonging to the “calcium ion binding”, “signaling receptor activity” pathways (**Figure 4** and **Supplementary Table 2**). A few up-regulated genes encoding E3 ubiquitin-protein ligases such as plant U-box (PUB) ubiquitin ligases PUB14-like (Psat6g113600), PUB22-like (Psat5g260880) and PUB23-like (Psat3g028280, Psat6g119200, Psat6g119080) proteins were found in our experiments (**Figure 4** and **Supplementary Figure 4**). Recent studies have revealed that PUB proteins are involved in the negative and positive regulation of defense responses against pathogens (Gonzaílez-Lamothe et al., 2006; Trujillo et al., 2008; Yamaguchi et al., 2017). Since in *Arabidopsis* E3 ubiquitin-protein ligases AtPUB12 and AtPUB13 are involved in the negative regulation of the AtCERK1/AtLYK5 chitin receptor complex through its turnover (Liao et al., 2017; Yamaguchi et al., 2017), the activation of these proteins in pea may also contribute to the transient desensitization of chitin-induced responses. However, this assumption needs to be verified in further experiments.

Active oxygen species (AOS) generated in response to stimuli and during development can function as signaling molecules in plants, leading to specific downstream responses. The gene Psat4g205560 encoding serine/threonine-kinase OXI1-like protein was shown to be induced after CO8-DA treatment (**Figure 4** and **Supplementary Table 2**). Expression of an *Arabidopsis thaliana OXI1* gene encoding a serine/threonine kinase is induced in response to a wide range of elicitors and other H_2_O_2_-generating stimuli (Rentel et al., 2004). OXI1 is required for full activation of the mitogen-activated protein kinases MPK3 and MPK6 after treatment. Moreover, the activation Psat3g011120 encoding putative respiratory burst oxidase-like protein A (RbohA) in CO8-9 treated roots may be related to H_2_O_2_ generation.

Receptor-like cytoplasmic kinases (RLCKs), which lack extracellular ligand-binding domains, comprise a major class of signaling proteins that regulate plant cellular activities. Among of them, the OsRLCK185 and OsRLCK176 function downstream of CERK1 in the microbial peptidoglycan and fungal chitin signaling pathways that mediate innate immunity (Ao et al., 2014; Wang et al., 2017; Yamaguchi et al., 2017). The homologous RLCK AtPBL27 in *Arabidopsis* participates in the activation of defense genes during response to chitin, peptidoglycan and their derivatives. The expression levels of Psat1g187600, Psat1g187560 transcripts encoding OsRLCK185/AtPBL27-like protein and Psat3g139680 encoding OsRLCK176-like protein were quite high in the CO8-DA-treated and control roots in our experiments, although they did not fall into the group of differentially expressed genes (**Supplementary Table 3**). Since these protein kinases may be related to endogenous intracellular signaling downstream of CERK1-like receptor, the LYK9, the role of pea AtPBL27/OsRLCK185-like and OsRLCK176-like protein kinases will be verified in our subsequent experiments.

An essential up-regulation of Psat3g028080 transcript encoding calcium-dependent protein kinase may be related to calcium signaling after CO8-DA perception. In addition, stimulation of the Psat7g118200 encoding glutamate receptor-like channel may be related to calcium influx into the cell as previous studies showed (Kwaaitaal et al., 2011). It demonstrates that CO8-DA elicitor might induce elevation in the concentration of free cytosolic calcium (**Figure 4** and **Supplementary Table 2**). Indeed, among differentially expressed genes a lot of calcium-binding proteins were found. As examples, the Psat5g257040, Psat3g061280, Psat2g004960 encoding calcium-transporting ATPase and Psat3g049400 encoding IQ1 calcium, calmodulin-binding protein were found to be differentially regulated. Moreover, the Psat0s3961g0080, Psat0s3961g0120, Psat5g084320, Psat0ss2585g0440, Psat4g090880, Psat3g200360 encoding EF hand calcium-binding family proteins were also up-regulated after treatment.

**Figure 5:**
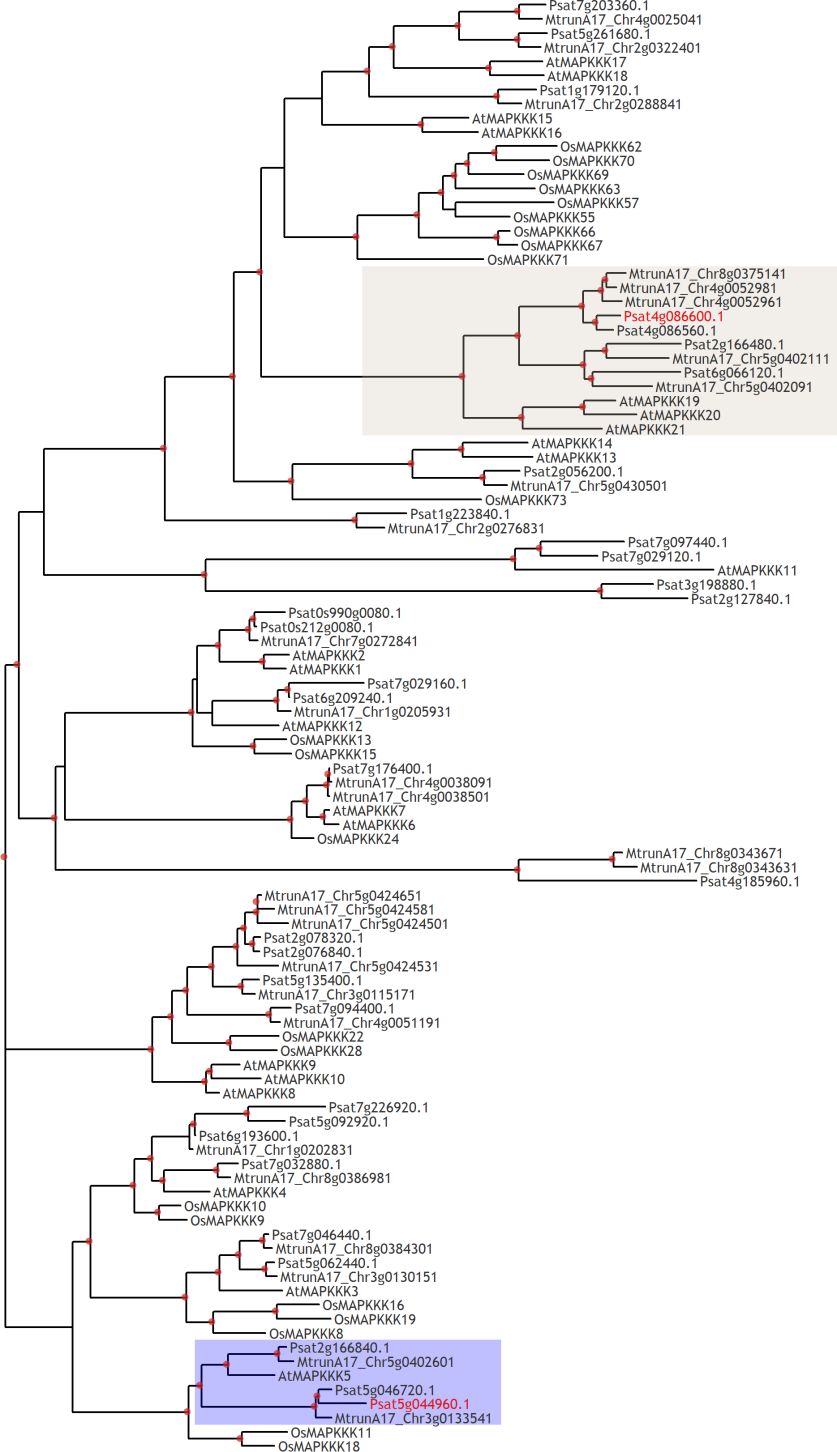
Phylogenetic tree constructed by the Maximum Likelihood method based on amino acid sequences of MAPKKKs from *Arabidopsis thaliana*, rice *Oryza sativa* and legumes: *Medicago truncatula*, *Pisum sativum*. Red dots indicate branch support more than 80 based on 1000 bootstrap replicates. Red labels show genome identifiers of PsMAPKKK5 (Psat5g044960) and PsMAPKKK20 (Psat4g086600).

Another type of intracellular regulators appearing as mitogen-activated protein kinase kinase kinases (MAPKKKs) such as Psat5g044960 encoding PsMAPKKK5 and Psat4g086600 encoding PsMAPKKK20 were revealed to be stimulated (**Figure 4** and **Supplementary Table 2**). These proteins showed a high level of homology with genes encoding MAPKKKs in *Arabidopsis*, rice, tobacco and model legume *Medicago truncatula* (**Figure 5**). Therefore, analysis showed that typical regulators of intracellular signal transduction pathways involved in triggering of plant responses *via* CERK1 receptors in *Arabidopsis* and rice may also be recruited in legume pea plants. Activation of several MAPKKKs indicates that these regulators might function redundantly in chitin/COs signaling. It is important to note, that pea PsMAPKKK5 protein showed the highest homology level with *Arabidopsis* AtMAPKKK5 and rice OsMAPKKK18, OsMAPKKK11 involved in chitin and COs perception (**Figure 5**) (Rao et al., 2010; Yamada et al., 2016; Kawasaki et al., 2017; Wang et al., 2017; Yamada et al., 2017). Another PsMAPKKK20 protein belongs to previously unstudied type of MAPKKKs. The AtMAPKKK5 and OsMAPKKK18, OsMAPKKK11 from *Arabidopsis* and rice were shown to be up-regulated in response to chitin/COs treatment and involved in subsequent phosphorylation of downstream MAPKK and MAPK signal regulators (Yamada et al., 2016; Kawasaki et al., 2017; Yamada et al., 2017). Since we also observed the up-regulation of *PsMAPKKK5* and *PsMAPKKK20*, this was in line with previous results.

### Searching the components of chitin/COs-triggered MAP kinase cascade activation

**Figure 6:**
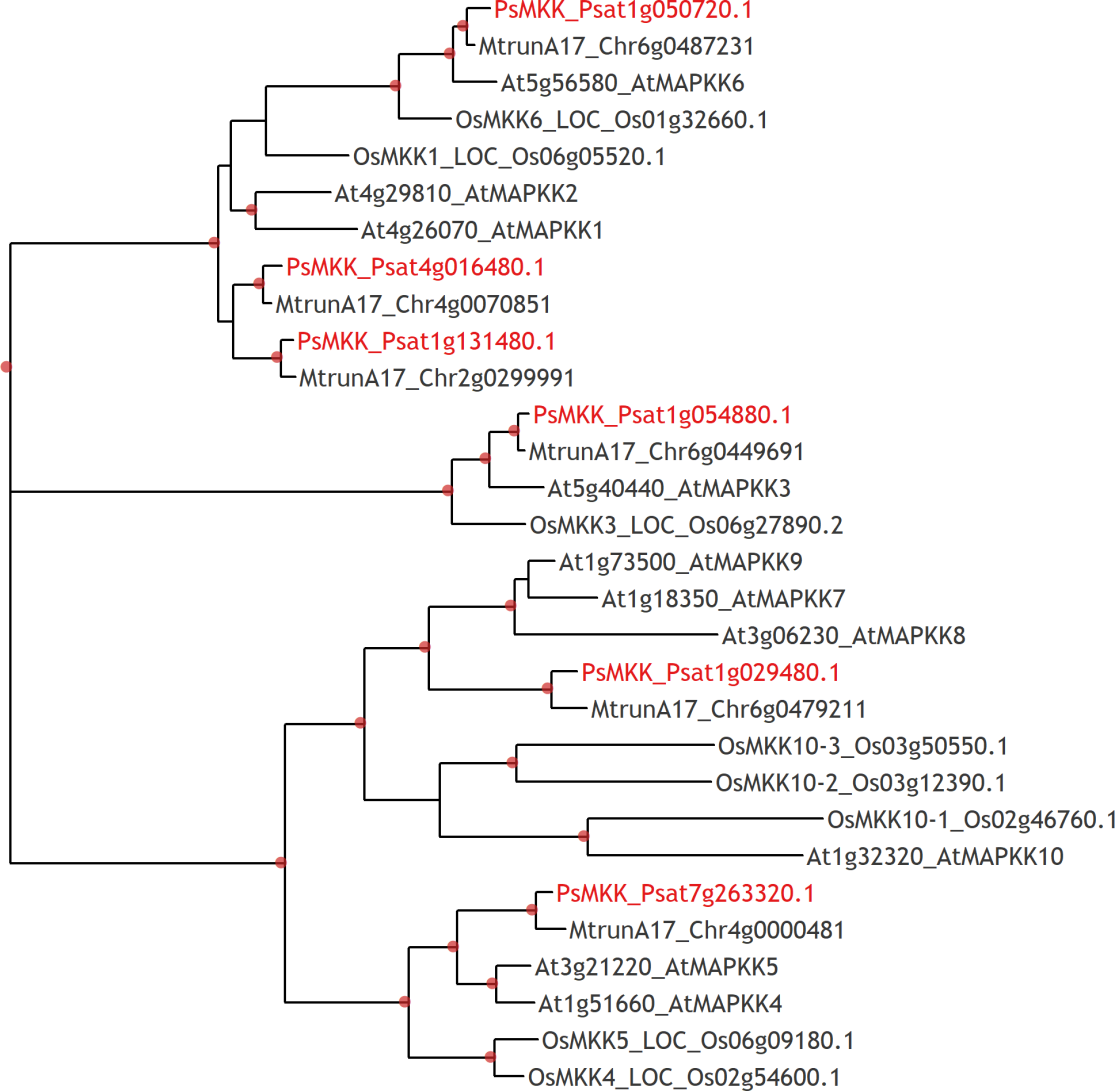
Phylogenetic tree constructed by the Maximum Likelihood method based on amino acid sequences of MAPKKs from *Arabidopsis thaliana*, rice *Oryza sativa* and legumes: *Medicago truncatula*, *Pisum sativum*. Red labels show genome identifiers of PsMAPKK6 (Psat1g029480), PsMAPKK2 (Psat4g016480), and PsMAPKK1 (Psat1g131480).

**Figure 7:**
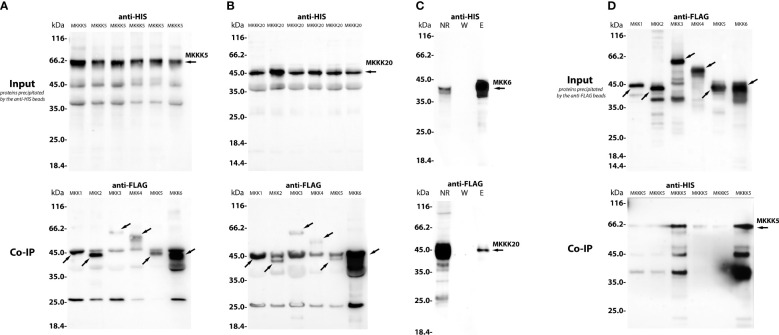
The co-immunoprecipitation of PsMAPKKK5, PsMAPKKK20 and PsMAPKK1, PsMAPKK2, PsMAPKK3, PsMAPKK4, PsMAPKK5, PsMAPKK6 from *P. sativum* cv. Finale. A heterologous protein expression was carried out in *Escherichia coli* C41, allowing high levels of expression of all proteins of interest fused to 6xHIS or 3xFLAG tag **(A–D)**. The lysates containing HIS-tagged and FLAG-tagged proteins were co-incubated with beads for 1 – 1.5 h on ice and then were loaded onto a µMACS column placed in the magnetic stand. After washing 3 - 4 times, the elution was done with a denaturing elution buffer followed by Western-blot analysis. In panels **(A, B)**, the HIS-tagged proteins (PsMAPKKK5, PsMAPKKK20) and FLAG-tagged proteins (PsMAPKK1, PsMAPKK2, PsMAPKK3, PsMAPKK4, PsMAPKK5, PsMAPKK6) were precipitated by anti-HIS magnetic beads **(A, B)**. In panel **(C)**, the PsMAPKK6 HIS-tagged protein and PsMAPKKK20 FLAG-tagged protein were also precipitated by anti-HIS beads **(C)**. In panel **(D)**, the FLAG-tagged proteins (PsMAPKK1, PsMAPKK2, PsMAPKK3, PsMAPKK4, PsMAPKK5, PsMAPKK6) and PsMAPKKK5 HIS-tagged protein were precipitated by anti-FLAG beads **(D)**. (NR – non retained fraction, W – washing, El – elution). The molecular weight of MKKK5 is about 65 kDa, while the weight of MKKK20 is about 45 kDa **(A, B)**. The molecular weight of MKK1 is about 45 kDa, MKK2 – 43 kDa, MKK3 – 65 kDa, MKK4 – 55 kDa, MKK5 – 43 kDa and MKK6 – 43 kDa **(C, D)**. The assays revealed the strong interaction between PsMAPKKK5 and PsMAPKK6 **(A)** as well as PsMAPKKK20 and PsMAPKK6 **(B–D)**, while both PsMAPKKK5 and PsMAPKKK20 showed only weak interactions with other PsMAPKKs.

To address whether PsMAPKKK5 and PsMAPKKK20 are involved in CO8-DA-induced defense activation, the searching of downstream targets such as MAP kinase kinases (MAPKKs) (**Figure 6**) was performed. To demonstrate direct interaction between MAPKKKs with their substrates, the MAPKKs, we expressed the full-length PsMAPKKK5 and PsMAPKKK20 in *Escherichia coli* as recombinant proteins fused to the N-terminus of 6xHIS (**Figures 7A, B**), and loaded them onto the columns with immobilized anti-6xHIS antibodies for co-immunoprecipitation (Co-IP). In the pea genome six genes encoding MAPKK were identified (**Figure 6**). All these six pea MAPKK such as PsMAPKK1, PsMAPKK2, PsMAPKK3, PsMAPKK4, PsMAPKK5 and PsMAPKK6 were expressed fused with 3xFLAG and used for Co-IP onto the columns with immobilized anti-6xHIS antibodies (**Figures 7A, B** and **Supplementary Figure 5**). Additionally, the Co-IP on the columns with immobilized anti-FLAG antibodies was also carried out for PsMAPKK1, PsMAPKK2, PsMAPKK3, PsMAPKK4, PsMAPKK5, PsMAPKK6 fused with 3xFLAG and PsMAPKKK5 fused with 6xHIS (**Figure 7D**). The Co-IP assays showed that both PsMAPKKK5 and PsMAPKKK20 were able to directly and strongly interact with PsMAPKK6 and showed only weak interaction with other PsMAPKKs (**Figures 7A, B, D**). The control experiments were also performed to test the non-specific binding of proteins with both type of beads (**Supplementary Figure 6**). Although we failed to detect it for almost all proteins, some non-specific binding was found for PsMAPKK6 on beads with immobilized anti-HIS antibodies (**Supplementary Figure 6**). To confirm the real interaction between two PsMAPKKKs and PsMAPKK6, the HIS-tagged PsMAPKK6 was also obtained in *E. coli* and tested with FLAG-tagged PsMAPKKK20 (**Figure 7C**). After Co-IP using anti-HIS beads, both proteins were found in eluted fraction. These results confirmed the interaction between tested proteins (**Figure 7C**). Therefore, our experiments showed the potential targets of two MAPKKKs during signal transduction in response to CO8-DA perception by pea plants.

**Figure 8:**
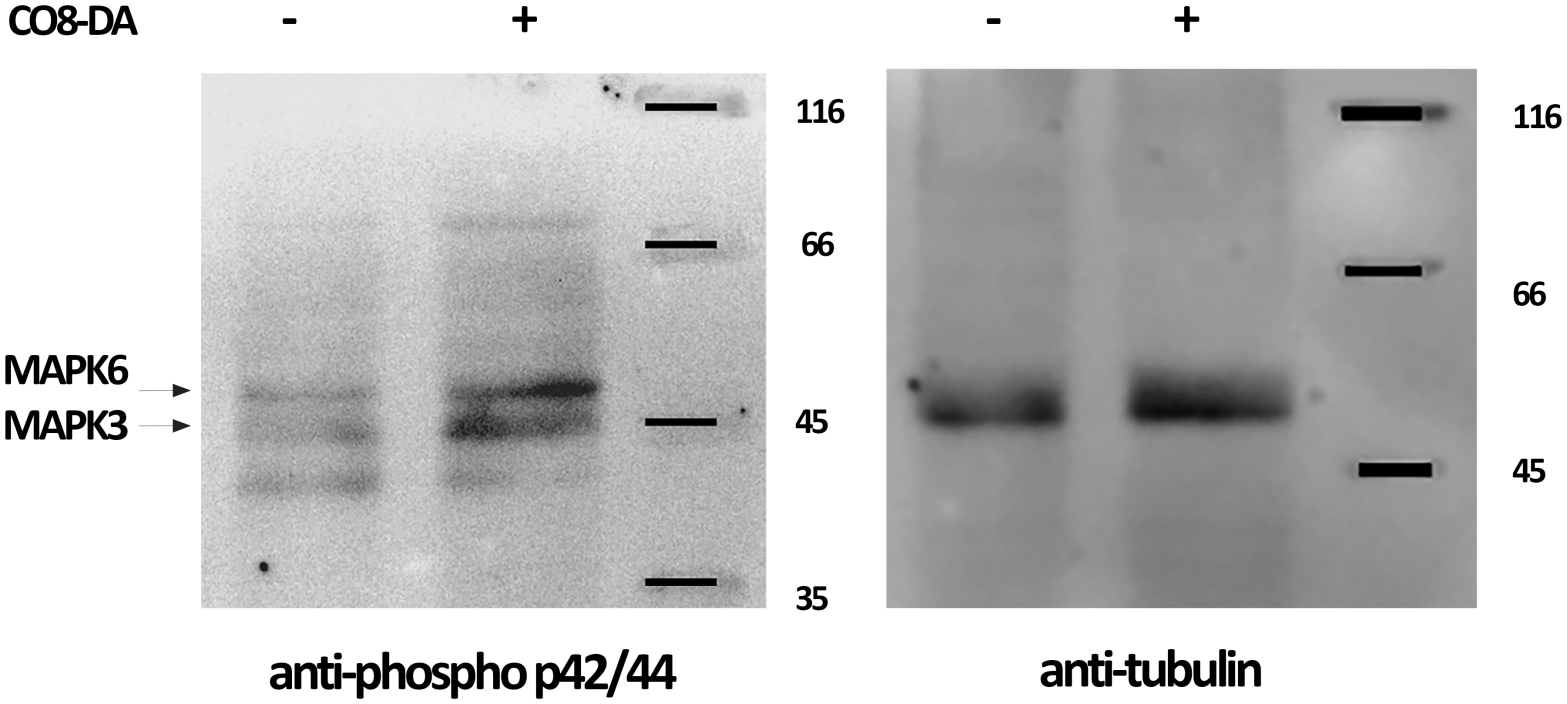
CO8-DA-induced MAPK3/6 phosphorylation in *Pisum sativum* cv. Finale. Plant treatment was done with 10^-5^ M CO8-DA (+) as elicitor for 15 min and 0.5x Jensen’s medium (-) as a control in glass jars. Western-blots were done to visualize the phospho-MAPK3/6 and tubulin bands using anti–phospho-p44/42 MAPK and anti–α/β-tubulin antibodies.

It was previously shown that the CERK1-like receptors in model legume plants *M. truncatula* (MtLYK9) and *Lotus japonicus* (LjCERK6) are able to activate MAPK3/MAPK6 after COs perception (Bozsoki et al., 2017; Gibelin-Viala et al., 2019). Indeed, using specific anti-phospho-p44/42 MAPK antibodies the phosphorylation of PsMAPK3/PsMAPK6 in pea in response to CO8-DA treatment was also revealed in our experiments (**Figure 8**).

### Analysis of involvement of MAPKKK in plant resistance to pathogens

**Figure 9:**
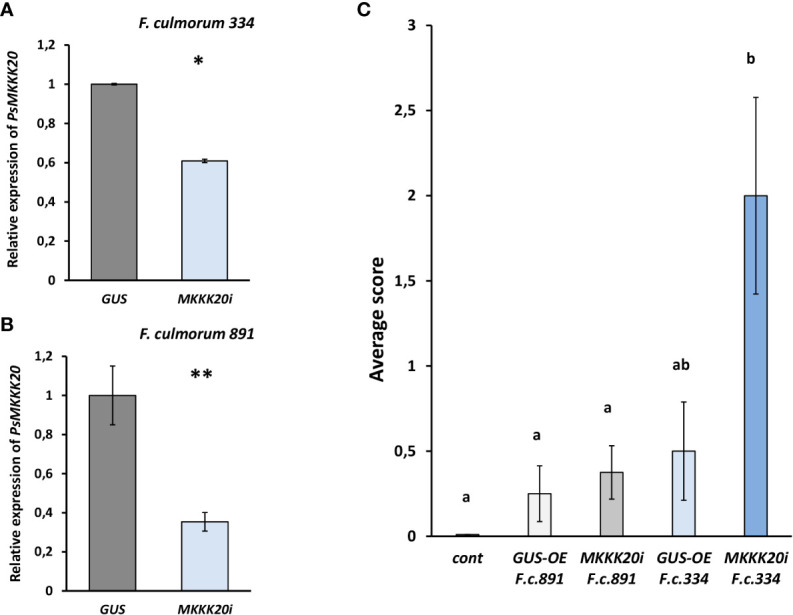
The effect of *PsMAPKKK20* gene suppression on resistance to the pathogenic fungus *Fusarium culmorum* highly aggressive 334 **(A)** and weakly aggressive 891 strains **(B)** in *Pisum sativum* cv. Finale plants. Composite plants with the *PsMAPKKK20* gene suppression in transgenic roots (*PsMAPKKK20-RNAi*) were compared with control plants with β-glucuronidase gene overexpression (*GUS-OE*). Analysis was performed two weeks after infection with *F. culmorum*. Approximately 50 - 60% suppression of the *PsMAPKKK20* gene in the transgenic roots of pea was revealed **(A, B)**. One-way ANOVA test was used to compare gene expression levels. The resistance to infection in transgenic roots of *PsMAPKKK20*-RNAi plants was significantly reduced in comparison to *GUS-OE* control **(C)**. Two biological replicates were analyzed for *P. sativum*, each containing 10 – 15 plants per variant. Totally about 40 and 30 transgenic fluorescent roots of *PsMAPKKK20*-RNAi and *GUS-OE* plants, correspondingly, were included in the analysis. Kruskal-Wallis test (R-studio) was used to compare infection scores in *PsMAPKKK20*-RNAi and *GUS-OE* variants. Values are means ± SEM. *, values with various letters are significantly different at P < 0.05; **, significant difference at P < 0.01.

To further verify the role of MAPKKKs in pea plant response to CO8-DA found in transcriptome assay, the RNAi based experiments were performed. We focused on gene encoding PsMAPKKK20, because it belongs to another type of MAPKKKs not previously studied in plants. To better understand the role of this signal component in plants, the suppression of *PsMAPKKK20* was done using RNA interference and showed to be around 50 – 60% in comparison to control plants transformed with *GUS* gene under 35S promoter (GUS-overexpression, GUS-OE) (**Figures 9A,B**). Infection of these composite plants with phytopathogenic fungi *Fusarium culmorum* 891 and 334 strains differing in aggressiveness showed increased susceptibility of *PsMAPKKK20-*RNAi pea plants to pathogens, especially to highly aggressive 334 fungal strain (**Figure 9C**). Therefore, the silencing of *PsMAPKKK20* by RNAi enhances susceptibility to infection. It suggests that stimulated CO8-DA elicitor treatment *PsMAPKKK20* can be involved in the regulation of pea plant resistance to pathogens.

## Discussion

Since our experiments were aimed at clarifying the molecular and cellular modes of action of COs in crop legume *P. sativum* L., we investigated the transcriptional changes in control and treated pea roots *via* RNA sequencing. Here we describe the pathways and mechanisms triggered by CO8-DA and operating in crop legume plant *P. sativum* L.

Taken together, our results point to a rapid and significant activation of pea genes involved in regulation of host defense responses, induced by CO8-DA, in accordance with previous findings for chitin-related compounds in *Arabidopsis*, rice, potato, tea (Chen et al., 2020; Lemke et al., 2020; Siriwong et al., 2021; Ichimaru et al., 2022; Ou et al., 2022). We have shown that a set of genes encoding specific enzymes involved in the biosynthesis of phytoalexins and other secondary metabolites was up-regulated in response to CO8-DA, that was in line with previous findings in other plants (Vasyukova et al., 2001; Khan et al., 2003; Zhang et al., 2020). The pea plant treatment with CO8-DA stimulated the synthesis of such components as lignins, reinforcing the cell wall. Similar effect of COs was previously shown in rice and tea (Siriwong et al., 2021; Ou et al., 2022).

At the same time, one of the most important aims during screening of differentially expressed genes may be searching for the specific genes responsible for plant resistance activation to pathogens. Since these genes may be the subject of direct genetic manipulations to increase plant crop stability. Indeed, some genes encoding disease resistance proteins such as Psat7g105600, Psat4g088560, Psat7g083880 were identified in our experiments as probable orthologs of genes previously found and well-studied in *Arabidopsis* (At5g15730, At5g66900, At1g18390). The information about homologous genes in *Arabidopsis* indicates their involvement in a positive regulation of resistance to biotic and abiotic stress responses. Therefore, these genes are potential targets for increasing legume plant resistance to pathogens.

Based on our transcriptomic analysis there is some evidence that CO8-DA elicitor may stimulate expression of genes encoding NBS-LRRs or NLRs, thereby increasing plant immune response. In susceptible plants, pathogens inject effectors into the host cell to prevent the immune response. In contrast, recognition of effectors by NLRs leads to ETI activation resulting in the “hypersensitive response” followed by death of the host cell and restriction of pathogen growth (Heath, 2000; Jones and Dangl, 2006). Recent studies showed that heterologous expression of the *NBS-LRR* genes may significantly enhance pathogen resistance in recipient plants (Li et al., 2018). It demonstrates that application of CO elicitors for stimulation of these genes may be considered as a promising tool for manipulation with plant resistance.

The results demonstrate that CO8-DA treatment may affect the hormonal status of pea plants. We have shown that ethylene mediated signaling pathway is up-regulated by CO8-DA. Moreover, a number of genes related to jasmonic acid (JA) and salicylic acid (SA) signaling events were found to be differentially regulated. Analysis of literature data suggests that these pathways are required for plant defense response development as it was shown previously for other plants (Jwa et al., 2002; Miller et al., 2017).

Calcium signaling plays a key role in plant responses to environmental stimuli. In *Arabidopsis*, stimulation of PRRs by MAMPs (flg22, elf18, chitin/COs) results in a rapid transient influx of extracellular Ca^2+^ into the cytosol, followed by the activation of receptor-like cytoplasmic kinases and mitogen-activated protein kinases (Ranf et al., 2011; Bigeard et al., 2015). Indeed, our results suggest the existence of a signaling pathway from CERK1-like receptor, the LYK9, to intracellular activation of calcium signaling and an MAPK cascade in response to treatment in pea *Pisum sativum* L. Perception of CO8-DA by pea plant induced the expression of genes related to regulation of calcium signaling in the cells. Among them, the gene encoding glutamate receptor-like channel and calcium-dependent protein kinase were found in our experiments. Here we found two MAPKKKs, the PsMAPKKK5 and PsMAPKKK20, which might function redundantly in the chitin/COs signaling pathway. At the next step, the PsMAPKK6 plays the most important role in the signal transduction pathway that is followed by PsMAPK3/PsMAPK6 activation. In accordance with this suggestion, we showed that *PsMAPKKK20*-RNAi knockdown may decrease chitin-induced defense marker activation and resistance to pathogenic *Fusarium* fungus.

These results were consistent with those in *Arabidopsis* and rice, where CERK1 elicitation was able to activate signal transduction, including calcium-regulated channels, receptor-like cytoplasmic kinases and mitogen-activated protein kinases, which might regulate the plant resistance. Previously, receptor-like cytoplasmic kinase AtPBL27 was reported to connect CERK1-mediated chitin/COs perception to MAPK cascade activation (AtMAPKKK5) in *Arabidopsis* (Shinya et al., 2014; Yamada et al., 2017). In rice, OsRLCK185 and OsRLCK176 might function redundantly and activate OsMAPKKK18 and OsMAPKKK11, rice orthologs of AtMAPKKK5, in the OsCERK1-induced chitin and peptidoglycan signaling pathways (Ao et al., 2014; Wang et al., 2017; Yamada et al., 2017; Yamaguchi et al., 2017). Therefore, our findings suggest that in pea the calcium signaling regulators and MAPK cascade activated by CO8-DA induce immune responses by phosphorylating a complex of downstream targets.

## Data availability statement

The data presented in the study are deposited in the NCBI repository, accession number GEO Submission (GSE218057).

## Author contributions

PK: Investigation, writing original draft preparation, methodology, and data analysis. OP: Investigation, heterologous protein synthesis, co-immunoprecipitation, library preparation. EK: Investigation, plant transformation, and library preparation. AB: Investigation, heterologous protein synthesis, and co-immunoprecipitation. AVD: Genome-based searching, analysis and graphic output of phylogenetic trees. SS: Methodology and cloning. AMD: Methodology and cloning. ED: Conceptualization, writing and editing, and supervision. All authors contributed to the article and approved the submitted version.
