# Supplementary material for: Transcriptomic analysis of pea plant responses to chitooligosaccharides’ treatment revealed stimulation of mitogen-activated protein kinase cascade
Source: Front Plant Sci. 2023 Mar 8;14:1092013. doi: 10.3389/fpls.2023.1092013 (PMC10030943; doi:10.3389/fpls.2023.1092013)
Supplement: Supplementary file 1 [file DataSheet_1.zip › Supplementary materials/Table S1.docx]

**Table S1**. List of primers

| PsMAPKKK20-RNAi_F_attB1 | AAAGCAGGCTTCCTGTTTCGGAGATGATTACACCT |
| --- | --- |
| PsMAPKKK20-RNAi_F_attB2 | AAGCTGGGTGCGGCGACATAAACAGTGGAGT |
| PsMAPKKK5_pRSETa_BglII_F | GGAGATCTGCATGACTCCTACTACTAACAAACC |
| PsMAPKKK5_pRSETa_EcoRI_R | GGGAATTCCTCAAAACATGGAAGTGGT |
| PsMAPKKK20_pRSETa_BamHI_F | GGGGATCCGATGGATTGGGTACGAGGAG |
| PsMAPKKK20_pRSETa_EcoRI_R | AGATAGTTGGATTAGTGTTAGGTGAGAATTCCC |
| PsMAPKK1_pRSETa_XhoI_F | GGGCTCGAGATGAAGAAAGGACGTTTAGATCC |
| PsMAPKK1_pRSETa_EcoRI_R | GGGGAATTCTTATATCGTTGCAAGTGTAGAACC |
| PsMAPKK2_pRSETa_BamHI_F | GGGGATCCATGAACAGAGGAAATTTTGGTCT |
| PsMAPKK2_pRSETa_EcoRI_R | GGGGAATTCTTATAAGGTAGCGAGTGGAGATCC |
| PsMAPKK3_pRSETa_SacI_F | GGGAGCTCATGAAGACAAAAACGCCATTGAA |
| PsMAPKK3_pRSETa_EcoR1_R | GGGGAATTCTCATTTGGGAAAATTTACAGGA |
| PsMAPKK4_pRSETa_XhoI_F | GGGCTCGAGATGCGAGAAAGAAATGAGATGAAGC |
| PsMAPKK4_pRSETa_HindIII_R | GGGAAGCTTCTAAGAAGAAAGTGGCCTTGGTG |
| PsMAPKK5_pRSETa_XhoI_F | GGGCTCGAGATGTCTGGTTTAGAGGAGTT |
| PsMAPKK5_pRSETa_PstI_F | AACTGCAGTTATTGGTTAATGTACAGTTCTTGT |
| PsMAPKK3_pRSETa_XhoI_F | GGGCTCGAGATGGCGCTCGTCCAC |
| PsMAPKK3_pRSETa_EcoRI_F | GGGGAATTCTTAACAACTATCCATTTCTTTACAC |
| Ubiquitin_PCR_F | ATGCAGATC/TTTTGTGAAGAC |
| Ubiquitin_PCR_R | ACCACCACGG/AAGACGGAG |
| PsMAPKKK20_PCR_F | CGCCGATGATACCCGAAGAT |
| PsMAPKKK20_PCR_R | CTACACGCGAAAAAGAACAAGAAT |
