# Supplementary figures and images for: Transcriptomic analysis of pea plant responses to chitooligosaccharides’ treatment revealed stimulation of mitogen-activated protein kinase cascade
Source: Front Plant Sci. 2023 Mar 8;14:1092013. doi: 10.3389/fpls.2023.1092013 (PMC10030943; doi:10.3389/fpls.2023.1092013)

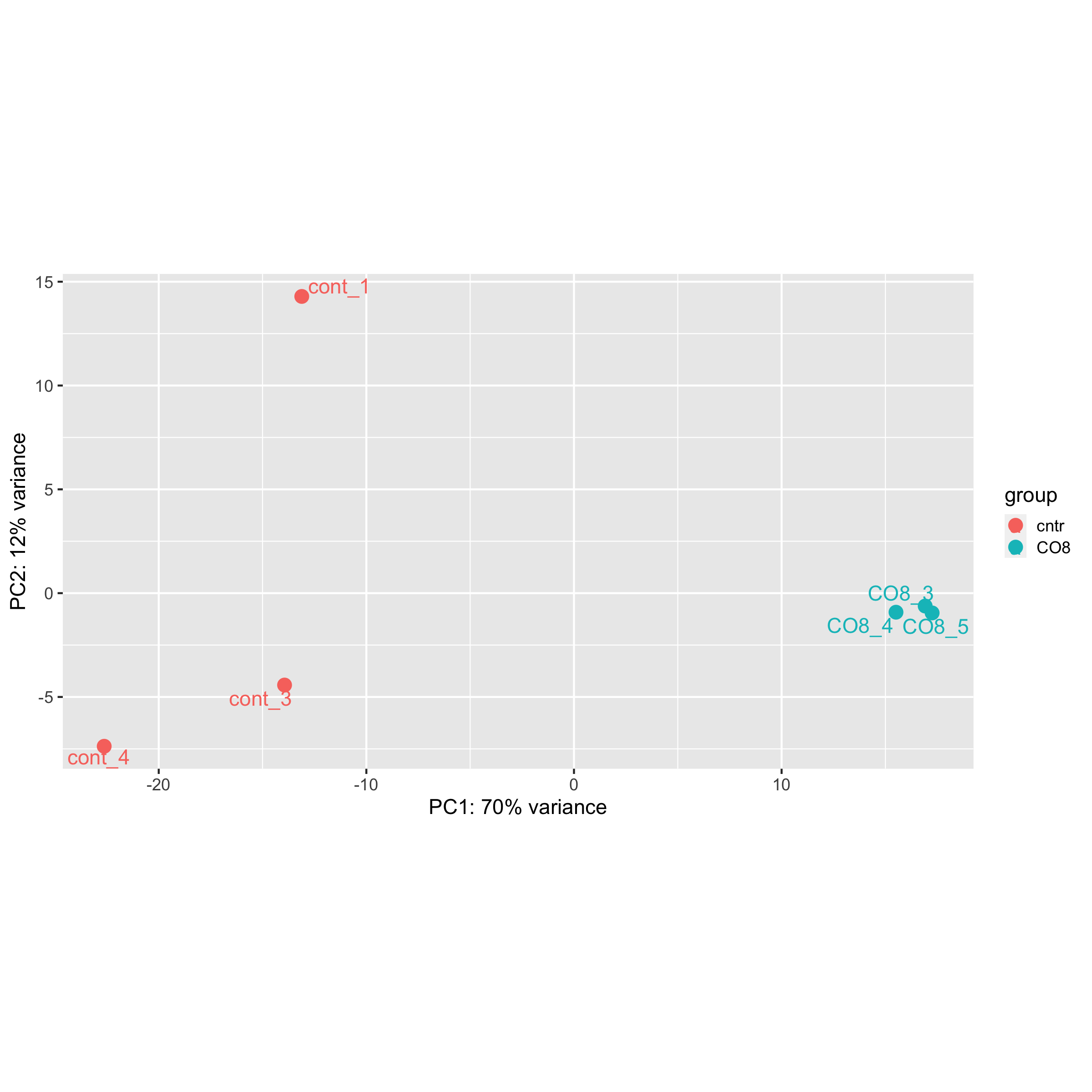

Supplement: Supplementary file 1 [file DataSheet_1.zip › Supplementary materials/Figure S1.png]

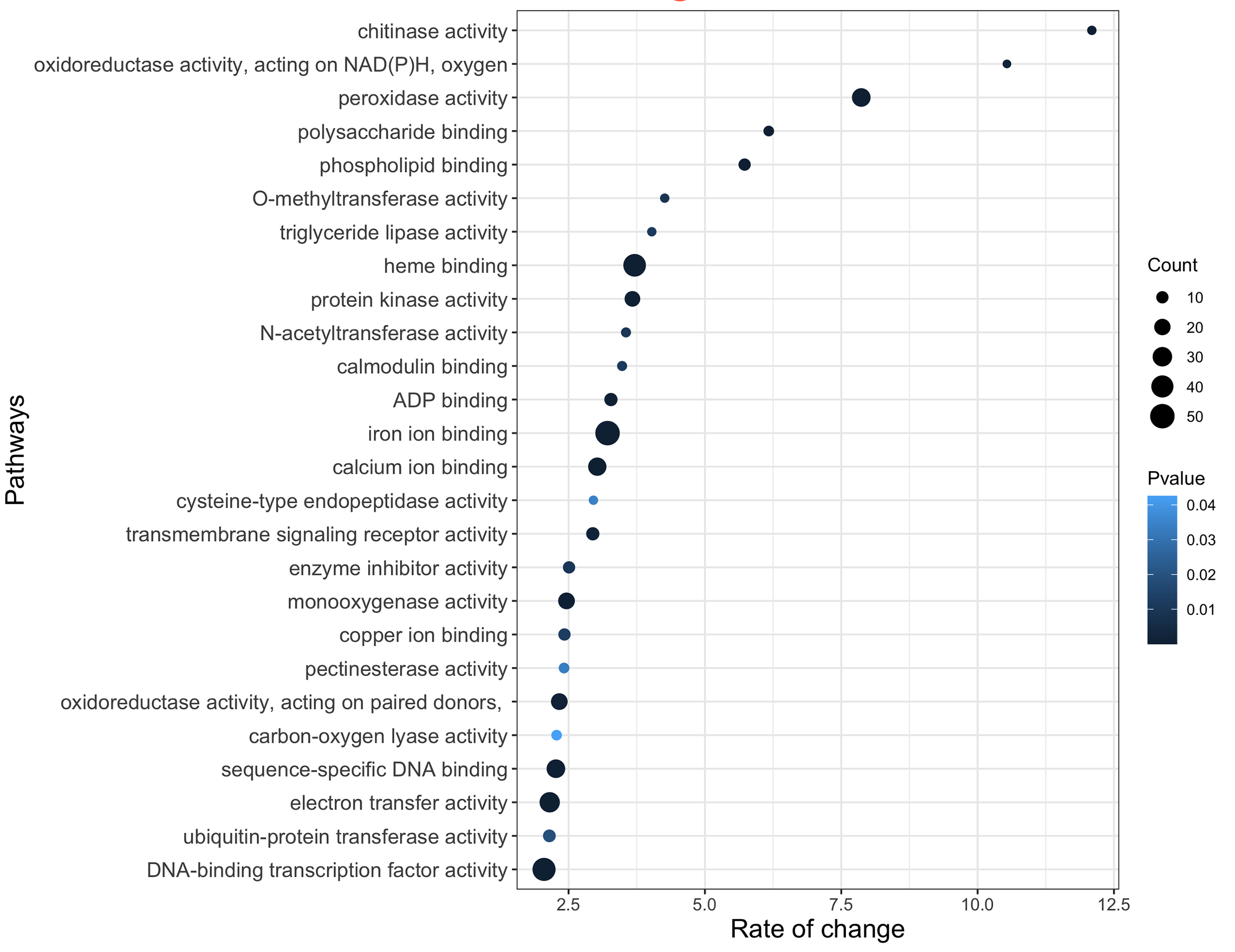

Supplement: Supplementary file 1 [file DataSheet_1.zip › Supplementary materials/Figure S2.png]

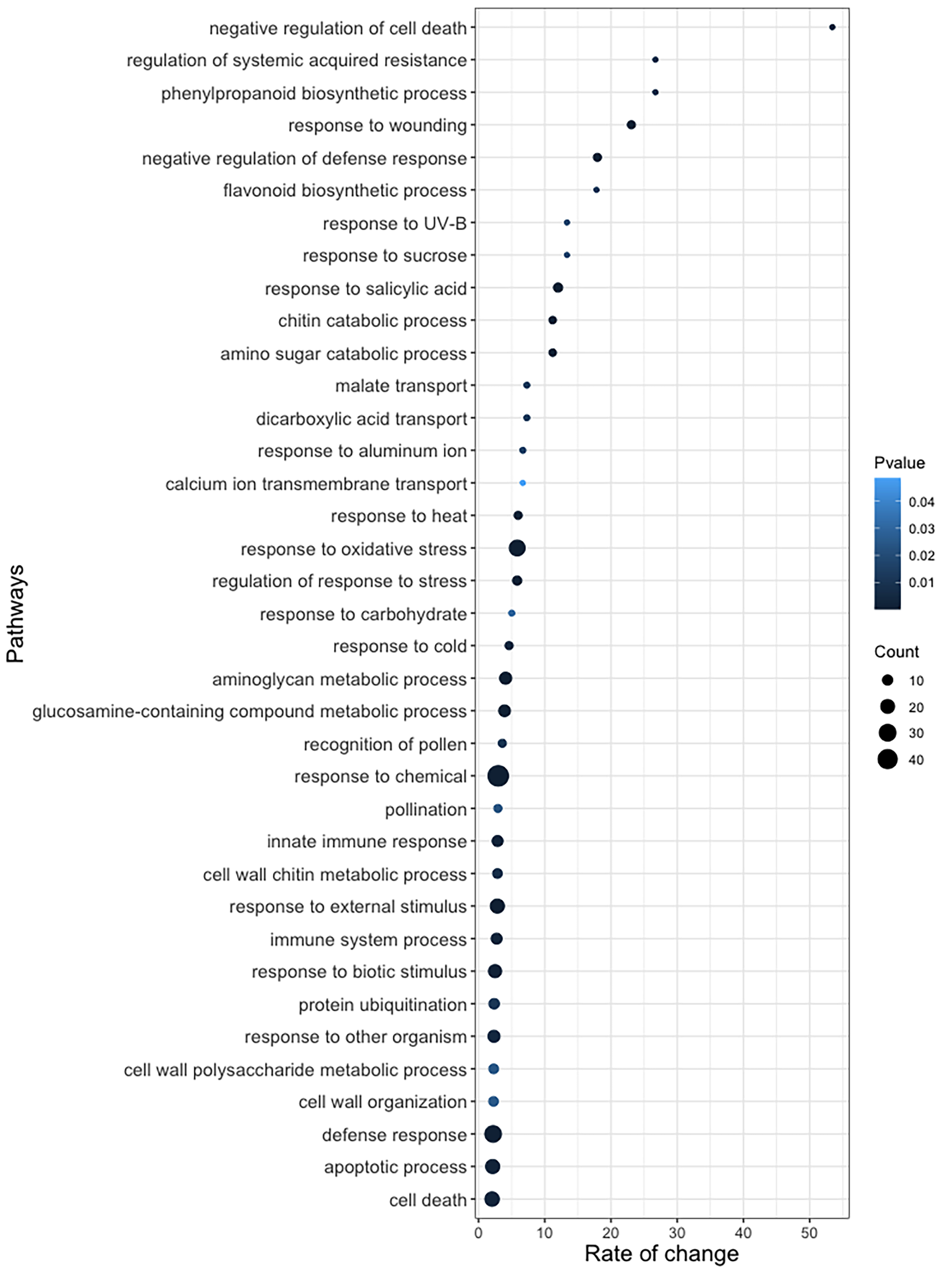

Supplement: Supplementary file 1 [file DataSheet_1.zip › Supplementary materials/Figure S3.png]

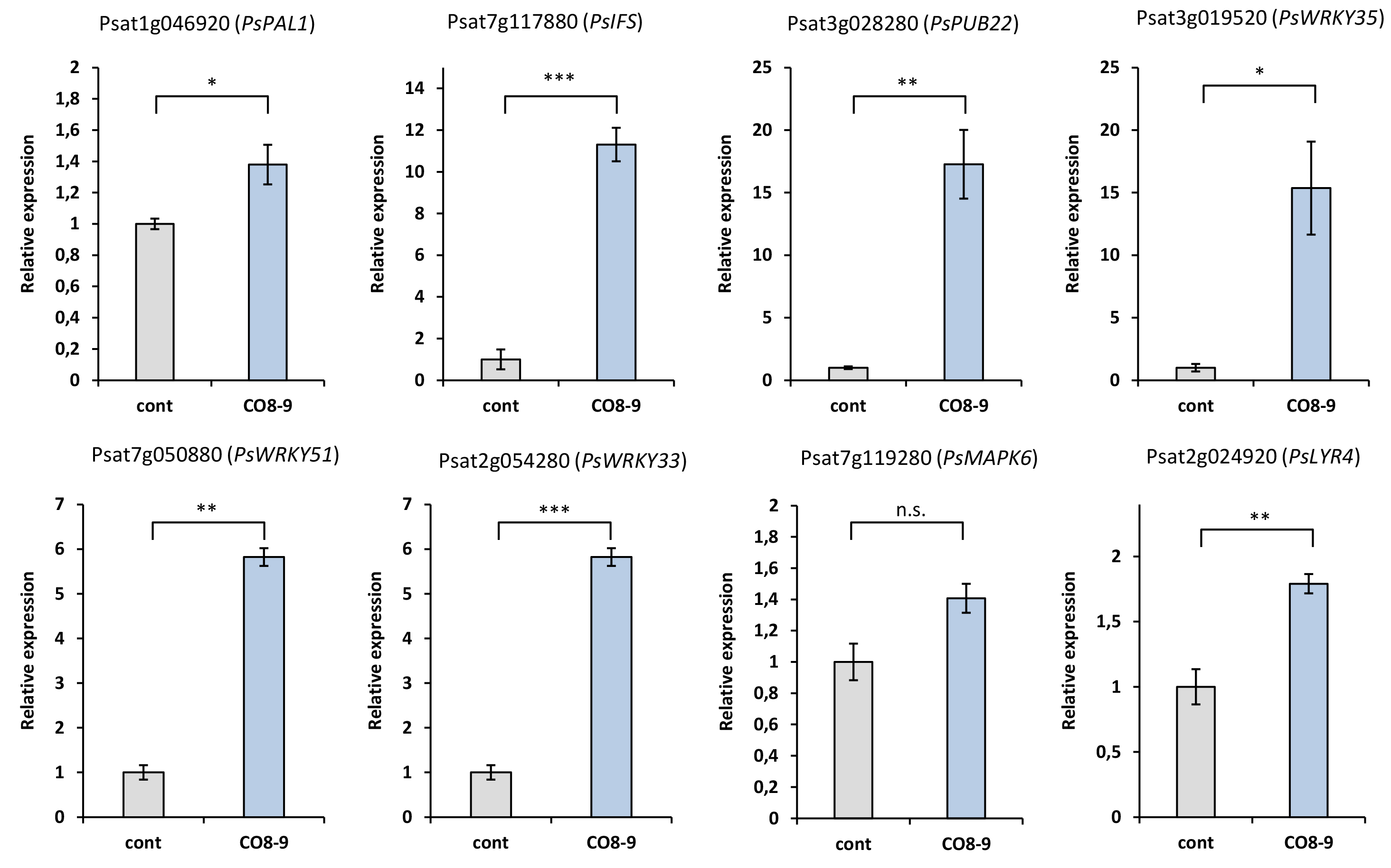

Supplement: Supplementary file 1 [file DataSheet_1.zip › Supplementary materials/Figure S4.tif]

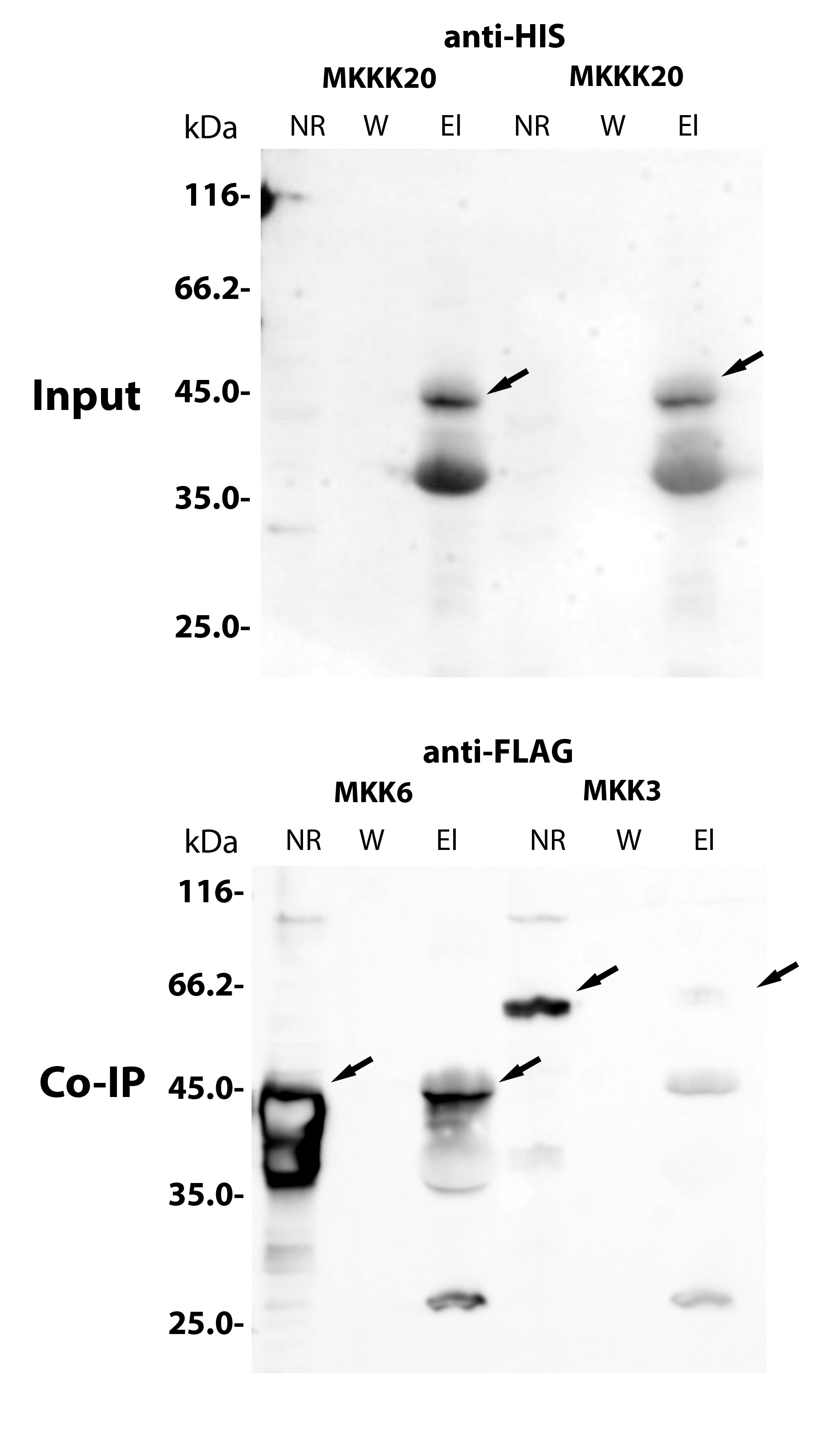

Supplement: Supplementary file 1 [file DataSheet_1.zip › Supplementary materials/Figure S5.tif]

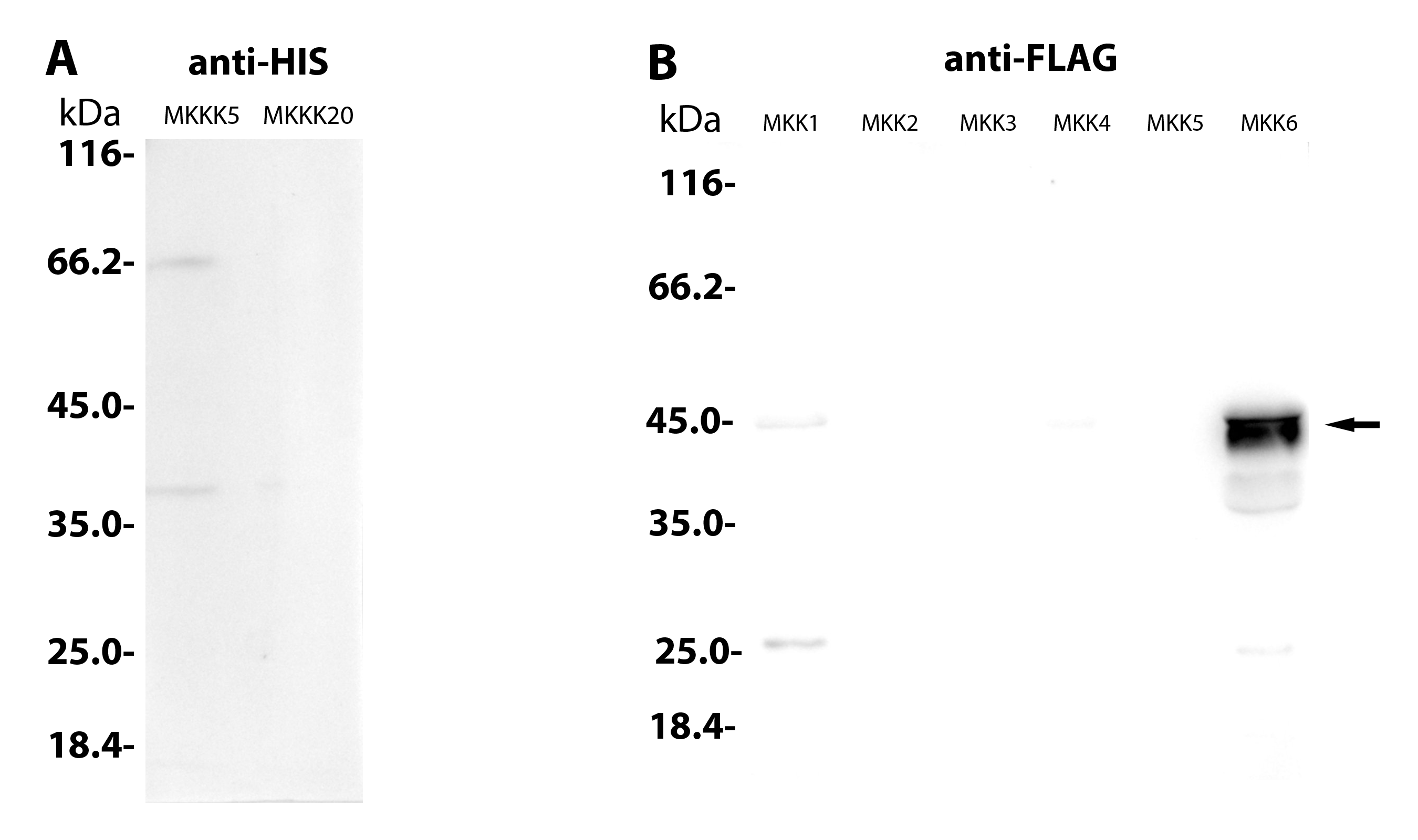

Supplement: Supplementary file 1 [file DataSheet_1.zip › Supplementary materials/Figure S6.tif]
